# Strategic Attributes and Organizational Performance: Toward an Understanding of the Mechanism Applied to the Banking Sector

**DOI:** 10.3389/fpsyg.2022.855910

**Published:** 2022-05-12

**Authors:** Honglei Tang, Zeeshan Rasool, Muhammad Sarmad, Ammar Ahmed, Umair Ahmed

**Affiliations:** ^1^School of Economics and Management, Huzhou University, Huzhou, China; ^2^NFC Institute of Engineering and Technology, Multan, Pakistan; ^3^School of Economics and Management, Shaanxi University of Science and Technology, Xi’an, China; ^4^Riphah School of Leadership, Riphah International University, Islamabad, Pakistan; ^5^Department of Management Sciences, MNS University of Engineering and Technology, Multan, Pakistan; ^6^Department of Management Sciences, Arab Open University, Manama, Bahrain

**Keywords:** strategic orientation (SO), organizational culture (OCU), organizational internal market orientation (IMO), organizational commitment (OCO), organizational performance (OP)

## Abstract

The study examines and theorizes the importance of strategic attribute constructs (strategic orientation, organizational culture, and organizational internal market orientation) as applied to Pakistan’s banking sector by identifying their roles in enhancing organizational performance and the mediating effect of organizational commitment. The current study adopted quantitative research designs and methods to determine structural relationships between the proposed constructs. A total of 10 hypotheses were tested underpinned by the Resource-Based View of the Firm and Social Exchange theories. The strategic attributes studied were significantly and positively related to, and enhance, organizational performance if the banks: (1) focus on strategic positioning, (2) fostering a strong organizational culture, (3) strategize internal marketing practices, and (4) boost organizational commitment. The findings supported the mediating role of organizational commitment between strategic attributes and organizational performance. This study contributes to existing literature and supports prior research while filling in gaps in the literature concerning developing countries.

## Introduction

A strong financial sector is considered a base for economic development in the current business environment. Moreover, to attain the uncertain and complicated requirements of the global market, the financial sector of any country must achieve strategic competitiveness by implementing extra efforts and tools (Acar et al., 2013). Typically, an effective banking system is one of the prime components of the financial sector throughout the world. Due to the globalization of banking operations, the focus on the banking sector has tremendously increased, especially in developing countries like Pakistan (Shahid et al., 2015). Therein, most banks have become more customer-centered because of high competition and technological advancement (Auerbach et al., 2012). Banks of any country are performing a dynamic part in the economic development. The banking sector provides the resources that lead the country to prosperity and development (Rotheli, 2010; Otto et al., 2012; George et al., 2013). In addition, banks perform intermediating roles between individuals or institutions who have funds in surplus and individuals or institutions who have deficit funds (Mahmood and Wahid, 2012). The roles and performance of the banks in the economy should be of concern to not only depositors, governments, and investors but also to others such as researchers and scholars.

The banking sector of Pakistan has passed through many phases due to economic and political issues (Butt, 2010). Undoubtedly, the growth has been noticed over the last few years. However, compared to its geographical counterparts, the country’s financial indicators have remained lower (Chhoangalia and Badshah, 2016). Sadly, only 13 percent of the population from age 18 and above has registered bank accounts in Pakistan. Similarly, the population interested in savings activity with formal saving accounts is only 3 percent. Finally, only 2 percent of the population has borrowed money from the banking sector, which undoubtedly represents the significant scope of growth for the banks to accelerate their business operations (Chhoangalia and Badshah, 2016). These indicators clearly show the relatively weak performance of the banking sector in Pakistan.

Fewer numbers of banks operate in Pakistan than in developed countries. For every 10,000 people, there is only a single bank branch in Pakistan. In developed nations, this number is 4,000 people. There are many reasons for the poor banking performance in Pakistan revealed by past studies, such as weak prevailing culture, poor corporate governance, high bank operating costs, low organizational commitment, employment issues, lack of employee morale and job satisfaction, increasing non-performing loans, and ineffective HR policies affected the performance of banking institutions adversely and resulted in financial losses (Badar, 2011; Chhoangalia and Badshah, 2016; Lopes and Kachalia, 2016). Furthermore, it has been emphasized that the banks should pay attention to their strategies to meet corporate challenges (Aslam, 2014).

In the pertinent literature, organizational performance refers to the organizational effectiveness to achieve its objectives (Bani-Hani et al., 2009; Abdalkrim, 2013; Choudhary et al., 2013). Furthermore, different organizations adopt different approaches for assessing organizational performance, as some measure from a financial viewpoint (e.g., Atoom et al., 2017; Birhanu et al., 2017), whereas others measure from non-financial viewpoints (e.g., Baird, 2017; Mehralian et al., 2017).

Past studies have described organizational performance as an essential concept that signifies the wide range of organizational activities (Hamdam et al., 2012; Ouakouak et al., 2014). Hence, there is a clear indicator of having effective and sustainable management strategies in the banking sector (Acar et al., 2013). Consequently, the present study attempted to examine the vitality of key strategic attributes like strategic orientation, organizational culture, organizational internal market orientation, and organizational commitment and their effect on organizational performance in the banking sector of Pakistan.

Strategic orientation predicts organizational performance and is considered an important strategic attribute (Weinzimmer et al., 2012). Strategic orientation is explained as, “the inclination of an organization to focus on strategic direction and proper strategic fit to ensure superior organizational performance” (Weinzimmer et al., 2012, p. 82). Effective strategic orientation by internal management can create many opportunities (Cheng et al., 2014). Accordingly, failure to adopt effective strategic orientation can seriously affect the achievement of higher performance targets (Aminu and Shariff, 2015).

However, multiple studies have been conducted to inspect the effect of strategic orientation on organizational performance, such as Weinzimmer et al. (2012) and Innocent (2015). Nonetheless, findings from the past studies are inconsistent (Han and Verma, 2012; Deshpandé et al., 2013; Ariyarathne, 2014; Choi and Yoon, 2015; Chahal et al., 2016; Song and Jing, 2017). Likewise, past studies have found a positive correlation between strategic orientation and organizational performance (Altindag et al., 2011; Eris and Ozmen, 2012; Theodosiou et al., 2012; Storey and Hughes, 2013; Jassmy and Bhaya, 2016). Previous research has indicated that strategic orientation has little or no effect on organizational performance and has recommended that future studies should consider strategic orientations in the context of developing countries (Cadogan, 2012; Altuntaş et al., 2013; Al-Ansaari et al., 2015; Obeidat, 2016). Thus, it is essential to evaluate the strategic orientation in Pakistani organizations, particularly in the banking sector for better performance prospects.

Denison (1990) defined organizational culture as the system of norms and values which is common among an organization’s employees and it determines the attitudes and approaches of organizational members toward confronting different problems in an organization. As a key strategic attribute, a healthy organizational culture is vital as it directly influences organizational performance (Weinzimmer et al., 2012; Storey and Hughes, 2013; Ahmed and Othman, 2017). Existing literature confirms both positive and negative effects of organizational culture on organizational performance (Chuang et al., 2012; Sokro, 2012). Regardless of the significance and potential effects of organizational culture, the relationship between organizational culture and organizational performance is immature, both empirically and theoretically (Sackmann, 2010; Weinzimmer et al., 2012). Thus, it is necessary to assess the importance of organizational culture in the banking sector of Pakistan to determine the discipline, acceptance of strategies, and focus on goals and their effect on organizational performance.

Human resources of any organization are considered the most valuable assets that can directly affect organizational performance (Kareem and Haseeni, 2015). Similarly, internal market orientation is also a crucial factor that is also used as an exchange of communication, means of resources, and other non-economic exchanges between employees and organizations (Lings and Greenley, 2010). Internal market orientation is summarized as the continuous activity done by the organization to fulfill the needs and wants of employees as the prerequisite to satisfying external customer needs and wants (Gounaris, 2008). Hence, internal market orientation primarily emphasizes internal customers (employees). Moreover, the nature and degree, and extent of internal market orientation in the banking sector have remained untouched in the existing literature (Kaur et al., 2008). Further research is therefore needed to empirically examine the internal market orientation of the organization and its effect on organizational outcomes (Lings and Greenley, 2010; Ferdous and Polonsky, 2011; Bhattarai et al., 2019; Jiang et al., 2020).

Organizational commitment is explained as an employee’s emotional attachment, identification with, and involvement in the organization (Jaworski and Kohli, 1993). Furthermore, some past studies have established a direct relationship between organizational commitment and organizational performance (Zincirkiran et al., 2015; Chai et al., 2016). Additionally, previous studies have highlighted the direct means of a constructive relationship between organizational culture and organizational commitment (Lim, 2010; Zavyalova and Kucherov, 2010). Joung et al. (2015) and Yu et al. (2016) also revealed the significant relationship between internal market orientation and organizational commitment. Hence, the relationships between strategic orientation (Liu and Fu, 2011), organization‘s internal market orientation (Kaur et al., 2008), organizational culture (Chuang et al., 2012), and organizational performance have never been studied in a single framework alongside the mediation of organizational commitment (Pinho et al., 2014).

The remainder of this article is classified as follows. In the next section, we review the existing literature. The literature review follows a discussion of research methods, unit of analysis, and subsequent results. The authors conclude by offering a number of recommendations for future studies, followed by discussion on the implications of the findings of this study.

## Literature Review

### Organizational Performance as Dependent Variable

Organizational performance refers to an appropriate organizational strategy (Parida et al., 2015) and is considered important in attaining organizational goals (Richard et al., 2009). The manufacturing sector has been studied (Verbeeten and Boons, 2009), as well as insurance (Lee and Yu, 2004), but little concern has been given to the banking industry (Elnihewi, 2015; Kamil and Nasurdin, 2016). Furthermore, the review of the literature showed that several experimental studies on organizational performance had been conducted in developed countries like United States and United Kingdom, and likewise in Asian countries like Malaysia and Singapore (Stede et al., 2006).

In the pertinent literature, past studies have identified the numerous predictors of organizational performance in different work settings, such as strategic performance measures (Pollanen et al., 2017), strategic hybrid orientation (Anees-ur-Rehman et al., 2017), corporate social responsibility (CSR) practices and innovation (Reverte et al., 2016), demographic and organizational factors (Wang et al., 2015), and entrepreneurial orientation (Brouthers et al., 2015). Few past studies have identified the predictors of organizational performance in the banking setting, such as entrepreneurial, market and strategic orientation (Innocent, 2015; Jassmy and Bhaya, 2016), service quality, innovation, and organizational commitment (Chai et al., 2016), and organizational learning and organizational culture (Aksoy et al., 2014).

### Underpinning Theory

The performance of organizations largely depends on how effectively the firm exchanges value with its members to utilize its resources effectively (Barney, 1991). Hence, based on such state of affairs, the review of pertinent literature reveals two basic theories to support the research model of underlying studies, such as the resource-based view (RBV) theory (Innocent, 2015) and the social exchange theory (SET) (Aldhuwaihi, 2013). The RBV assumed that an organization‘s internal properties are the basis of its success. These properties comprise of organizational capabilities and organizational resources (Bakar and Ahmad, 2010). The theory of RBV, typically shortened as RBV or RBT, is a popular theory that is extensively referred to and cited in microeconomics, strategic management, and other related fields, most particularly in the studies of organizational performance (Newbert, 2008). Further, the RBV serves as a major tool of the organizations to boost organizational performance and gain competitive advantage by focusing on the organizational resources. The theoretical lens of RBV has affirmed a relationship between organizational resources, competitive advantage, and organizational performance (Bertram, 2016).

Foa and Foa (2012) explained that the SET of resources resulted in positive and negative occurrences that the organizations must consider. In addition, SET assumes that the association between the organization and its employees involves both economic and social exchanges that encourage both organizational counterparts to perform the voluntary behavior as well as formal job obligations (Cropanzano et al., 2017). The SET is a persistent and widely used conceptual framework grounded on a very objective ideology. At different times, numerous essential topics related to organizational behavior have been studied using the lens of SET (Cropanzano et al., 2017). For instance, organizational commitment (Bishop et al., 2000), and organizational citizenship behavior (Cropanzano et al., 2017).

Thus, for this study, besides RBV, the SET appears as a supporting underpinning theory by demonstrating the organizational performance as a result of organizational culture, strategic orientation, organizational internal market orientation, and organizational commitment by means of an exchange between the organization and its employees. The SET has enough implications for the managers to increase employees’ trust and satisfy the mutual relationship between the organization and employees. Therefore, businesses should invest in the economic and social prospects of employees, which in return will motivate employees to invest their energies for organizational success.

### Relationship Between Strategic Orientation and Organizational Performance

The competitive position of an organization is strengthened through organizational strategies (Wheelen and Hunger, 2010). In the existing literature on organizational behavior, strategic management, and strategic marketing, strategic orientation is considered very critical in an organization because it determines the organizational success or failure (Weinzimmer et al., 2012). Typically, studies have linked strategic orientation with performance prospects (Ardito and Dangelico, 2018; Adams et al., 2019; Otache, 2019; Azaj et al., 2020). However, empirical studies on the topic of the relationship between organizational performance and strategic orientation (as a unidimensional construct) are very few in the ground of strategic management (Weinzimmer et al., 2012; Ahmed and Othman, 2017). Prior studies revealed that organizational performance was not affected by strategic orientation, which supports the findings of researchers such as Deshpandé et al. (2013), Al-Ansaari et al. (2015), and Obeidat (2016), even though it is also claimed that strategic orientation has a direct relation with organizational performance (Franczak et al., 2009; Eris and Ozmen, 2012). The reason behind this contradiction may be related to the fact that most of the previous studies mentioned relied upon altered measures of both strategic orientation and organizational performance (Liu and Fu, 2011). However, existing studies still have some deficiencies (Song and Jing, 2017). According to Adams et al. (2019), a substantial gap exists in researchers‘ knowledge and comprehension of how organizational capabilities can be leveraged to improve end results. Based on these, the present study attempted to test the following:

**H1:** Strategic Orientation has a positive effect on Organizational Performance.

### Relationship Between Organizational Culture and Organizational Performance

An organization’s culture affects the way it operates, enhances its staff abilities, and boosts its performance (Wu et al., 2011). Hatch and Zilber (2011) defined as the common beliefs and values that join the members of an organization. Concerning the above definition, culture is an essential element of an organization and differentiates it from the other organizations in the marketplace. Organizational culture holds paramount importance for any business entity (Yu et al., 2018). At the beginning of the 1980s, researchers started finding a strong contribution of shared values in organizations, resulting in higher organizational performance. Moreover, it has been widely emphasized in the literature of organizational studies that organizational culture extensively affects organizational performance because strong organizational culture is the key success factor for any organization (Weinzimmer et al., 2012; Pinho et al., 2014). But another researcher Lee (2006) found unpredictable results concerning the link between culture and organizational performance. Further, other empirical studies have supported the impact of organizational culture on for-profit organizations (Jaskyte, 2004; Hartnell et al., 2011). In connection to recent past studies such as Oyemomi et al. (2019) have landed support to organizational culture and performance link whereby Imran et al. (2021) reported mixed results in this regard. Thus, the relationship between organizational culture and organizational performance appears to be varying (Han and Verma, 2012; Uzkurt et al., 2013; Sylvie, 2016). This discussion has led to the development of the following hypothesis:

**H2**: Organizational Culture has a positive effect on Organizational Performance.

### Relationship Between Organizational Internal Market Orientation and Organizational Performance

The importance of human resource has been recognized, particularly in the service industry, which led to the enlargement of internal marketing orientation and/or internal marketing in the organization (Lings and Greenley, 2005). The term ‘human resource’ has been used to refer to the valued asset of the organization (Gul et al., 2012). This human factor is also considered very important and can directly affect organizational performance (Kareem and Haseeni, 2015). Likewise, Theodoridis and Panigyrakis (2011) found a positive relationship between internal marketing practices and organizational performance in the Greek retail market context. Similarly, Bhattarai et al. (2019) recently also reported significant relationship between the two. In addition, internal market orientation is also found promising in boosting pro-environment performance goals in the recent past (Jiang et al., 2020), thus highlighting it strategic significance. Although significant research has been conducted on internal marketing orientation, differences in the conceptualization in the pertinent literature have created boundaries for examining the impact of internal marketing orientation on organizational performance. Several studies have investigated the internal marketing orientation and its relationship with different approaches (Ferdous and Polonsky, 2011; Carlos and Rodrigues, 2012; Martin and To, 2013; Olusegun et al., 2020). As per Bhattarai et al. (2019), there is a constant need to assess the significance of market instruments to understand their impact on the businesses. Therefore, the following hypothesis is tested:

**H3:** There is a relationship between organizational internal market orientation and Organizational Performance.

### Relationship Concerning Strategic Orientation, Organizational Culture and Organizational Internal Market Orientation With Organizational Commitment

Numerous studies have begun examining the use of HR strategic orientation and found significant results affecting the organizational outcomes such as organizational commitment and performance (e.g., Kim and Kang, 2011; Chow et al., 2013; Darwish et al., 2013). Choi and Yoon (2015) stated that the highly strategically oriented HR functions influence organizational outcomes through employee commitment. Ismail (2016) examined the impact of training on affective commitment and considered two facets of strategic orientation, namely, learning goal orientation and performance goal orientation as moderators. Organizational culture is considered an important tool for the development of employee job satisfaction and organizational commitment (Silverthorne, 2004). Similarly, Lok and Crawford (2004) identified the organizational culture as an ancestor of organizational commitment, whereby Wu and Chen (2018) have reported leadership as a key ingredient for organizational commitment. Accordingly, organizational culture is found instrumental in predicting organizational commitment (Soomro and Shah, 2019; Pham Thi et al., 2021) whereby authors have indicated its vitality for businesses to achieve their goals and objectives across industries other than banks.

Notably, earlier research has recognized that internal marketing significantly affects organizational commitment (Lings and Greenley, 2005). The successful implementation of the internal marketing orientation is converted into positive employee results such as organizational commitment, work motivation, job involvement, and employee satisfaction (Ferdous and Polonsky, 2011). In contrast, lack of employee commitment can result in poorer performance and higher operating costs. Awwad and Agti (2011) conducted a study in the Jordanian banking sector and found the direct influence of internal market orientation on organizational commitment, organizational citizenship behavior, and market orientation. Khan (2012) studied Pakistani service organizations and indicated that internal marketing orientation predicted organizational citizenship behavior, organizational commitment, and market orientation. Gooshki et al. (2016) revealed that market orientation, organizational commitment, and organizational citizenship behavior are affected by internal marketing. In line with the RBV literature, internal marketing orientation is also considered as a unique organizational resource (Barney, 1991; Fang et al., 2014). Although recent literature has confirmed the importance of strategic orientation (Adams et al., 2019; Sahi et al., 2020) toward organizational prospects, its impact on organizational commitment appears to have been overshadowed, thus requiring further empirical attention. As a result, the following hypotheses are developed:

**H4:** Strategic orientation has a positive effect on organizational commitment.
**H5**: Organizational culture has a positive effect on organizational commitment.
**H6:** Organizational internal market orientation has a positive effect on organizational commitment.

### Organizational Commitment and Organizational Performance

How to get the best effort out of employees is a vital question for every employer and manager to know. History explains worker performance as employee commitment (Ali et al., 2010). In his study, Owens (2006) also found that committed employees are a great source of higher organizational performance and reduced employee work issues. Effective and valuable organizational commitment is always a result of employee core behavior, including turnover (Addae et al., 2006). A study in the healthcare sector also indicated the significance of organizational commitment to boost performance (Berberoglu, 2018) followed by Oyewobi et al. (2019) in the construction sector. Overall, it can be inferred from the above discussion that the relationship between these two (organizational commitment and organizational performance) is ambiguous and needs to be improved. Despite the importance (Wang et al., 2020), there is still a lack in the literature concerning the relationship between organizational commitment and organizational performance. Moreover, to what length OC can translate in the same way in the banking sector of Pakistan is another contextual gaps requiring empirical attention. Thus, the above arguments led to the development of the hypotheses mentioned below:

**H7:** Organizational commitment has a positive effect on organizational performance.

### Organizational Commitment as Mediator

In this study, organizational commitment is considered as the mediator between strategic attributes and organizational performance. In the pertinent literature, some of the previous studies revealed that organizational commitment acts as a potential mediator (Choi and Yoon, 2015; Omira, 2015; Kim et al., 2016; Majid et al., 2016; Hendri, 2019; Peng et al., 2020), while several studies found that organizational commitment does not mediate (Awwad and Agti, 2011; Zaman et al., 2012). In parallel, a recent study from Malaysia also reported no mediating effect of organizational commitment (Lim et al., 2017). Resultantly, these findings are raising the need for scholarly attention for confirmation.

Furthermore, it was noted that studies on organizational commitment were carried out in different work settings such as the casino industry (Kim et al., 2016), public organizations (Omira, 2015; Majid et al., 2016), local and multinational companies (Phetkaew, 2015), manufacturing (Tarigan and Ariani, 2015), and hotel industry (Hemdi and Rahim, 2011; Hamiza, 2014). However, limited studies have investigated the organizational commitment in the banking sector (Abdullah and Ramay, 2012; Hashmi and Naqvi, 2012). Likewise, prior empirical studies revealed the important mediating role of organizational commitment among organizational constructs (Rodrigues and Pinho, 2010; Pinho et al., 2014). Therefore, this study proposed the following mediating hypotheses.

**H8:** Organizational commitment mediates the relationship between strategic orientation and organizational performance.
**H9:** Organizational commitment mediates the relationship between organizational culture and organizational performance.
**H10**: Organizational commitment mediates the relationship between organizational internal market orientation and organizational performance.

Based on the development of the 10 hypotheses discussed above the following research model is developed:

## Methodology

### Population, Study Area, and Sample Size

The State Bank of Pakistan has classified the banks into three main categories based on their market share of deposits. Those with a market share greater than 6 percent are categorized as large banks. Accordingly, banks having a market share ranging between 3 and 6 percent are called medium banks, and those with less than 3 percent are called small banks (Chhoangalia and Badshah, 2016). There are six large banks (also called big banks) in Pakistan, which includes Habib Bank Limited with 14.1 percent market share, National Bank of Pakistan with 13.1 percent, United Bank Limited with 8.6 percent, Allied Bank Limited with 7.6 percent, MCB Bank Limited with 7.3percent, and Bank Alfalah Limited with 6.6 percent market share. These large banks have attracted much scholarly attention (Ahmed et al., 2017, 2019), as they make up almost 60% of total assets, deposits, and branches while contributing more than 70% of the total profitability of the entire Pakistani banking sector. By any measure, these six banks are the dominant banks in Pakistan (Chhoangalia and Badshah, 2016).

The current study adopted a quantitative research design to determine the structural relationships among the proposed constructs. The present study implemented the probability sampling design, because of the necessity to generalize the conclusion of the present study. In population, 1,786 bank branches of the six large banks of Pakistan, the study targeted the branches located in the country‘s major cities, including Karachi, Lahore, Peshawar, Quetta, and Islamabad. Bank branch managers were selected as the unit of analysis for the current study since they are subject to strive to implement organizational strategies and enhance the branch performance, which ultimately contributes to the organizational performance. Accordingly, after top management, bank branch managers are considered the most knowledgeable and well-versed. The data collection was approximately 7 months to ensure a healthy response rate.

According to this classification, the current study’s population was the bank branch managers of the six large banks of Pakistan across the five regional capital cities (PBA, 2016). It is commonly considered that each bank branch has one branch manager, who is accountable for the performance and success of the branch, and this is the unit of analysis for the current study.

The sample size was first determined through G*Power 3.1.9.2 software (Faul et al., 2009), giving a minimum sample size of 119, which is the basic requirement to deal with the partial least square structural equation modeling (PLS-SEM). Later, based on the population, the sample size was also determined through the Krejcie and Morgan (1970) approach and resulted in 317 as the sample size. Finally, a total of 260 bank branches responded to the survey, which represents an excellent response rate of 82%.

### Measurement Items

In this study, the independent variables are strategic orientation, organizational culture, organizational internal market orientation, the dependent variable is organizational performance and the mediating variable is organizational commitment.

In the current study perspective, organizational performance is the level of bank performance (increase/decrease) in financial and non-financial performance indicators. Organizational performance consists of two dimensions: financial performance (objective) and non-financial performance (subjective) measures which were measured through a 20-item scale. The organizational performance scale was adapted from Ringim (2012) and used in previous studies to determine bank performance (Elnihewi, 2015). In the present study, bank branch managers (respondents) were asked to rate their bank performance over the last 3 years, indicating the extent of perceived organizational performance across 20-items.

This study measured the strategic orientation as a unidimensional construct using 6-items and the scale was adapted from Weinzimmer et al. (2012). Also, this scale was used in previous studies to determine the strategic orientation of commercial banks (Innocent, 2015).

The current study adapted the 18-items of Denison (2000) to determine the effect of the organizational culture on organizational performance in the Pakistani banking sector. Considering these organizational culture items into practice, Al-Swidi (2012) used the similar Denison theory effectively to measure the organizational culture in the Yemeni banking sector. Therefore, researchers are encouraged to follow the similar 18-items of the scale to measure the organizational culture in the banking sector.

To measure internal market orientation behavior, the current study adapted a 4-item scale from Lings and Greenley (2005). Accordingly, a 7-item scale by Jaworski and Kohli (1993) was adapted and consistent with the previous studies to measure managers’ perception of organizational commitment (Kim et al., 2005; Rodrigues and Pinho, 2010; Pinho et al., 2014). The current study used the five-point Likert scale ranging from 1 as strongly disagree to 5 as strongly agree.

To pre-test the current study’s questionnaire, valuable suggestions were taken from the three faculty members of the College of Business from Universiti Utara Malaysia, having professional business experience and research expertise. The faculty members were requested to assess the survey instrument’s quality regarding its face validity relating to questionnaire wording (Yaghmale, 2009). Furthermore, valuable suggestions were also taken from three bank managers (potential respondents). Based on their feedback regarding simplicity, formatting, and clarity of ambiguous statements, improvements were made in the items. These improvements were necessary to ensure the high response rate achieved in this study.

### Data Analysis Process

Partial least squares (PLS) path modeling was used in the present study to verify the measurement and structural models. This technique has attracted much scholarly attention in the recent past (e.g., Darwish et al., 2021; Ibrahim et al., 2021). A measurement model described the validity and reliability of the constructs, whereas a structural model was used to carry out bivariate correlation analysis and regression analysis to simplify relations and their effects among constructs in this study. More importantly, the PLS algorithm and bootstrapping were used to find out the mediating effects of organizational commitment on the relationship between strategic orientation, organizational culture, organizational internal market orientation, and organizational performance relationships. Multivariate assumptions tests were also performed in this study, such as assessing missing values, outliers, normality test, and multicollinearity test.

### Common Method Variance

The current study implemented numerous procedural measures to reduce the effects of common method variance, as recommended by Podsakoff et al. (2012). First, the assurance was given to all the respondents (bank branch managers) that their responses were to be treated with high confidentiality. Second, the researcher removed the ambiguous items (used simple language) and used Harman’s single-factor test to investigate the common method variance in the current study. Based on Podsakoff et al. (2003), all the indicators of the current study were subjected to principal components factor analysis because the output of unrotated factors was analyzed to determine the number of factors required to account for the variance in the indicators.

The findings of the analysis generated one factor, explaining a cumulative of 35.409% of the variance, with the first (largest) factor explaining 19.475% of the total variances, which was less than 50% (Kumar, 2012). Also, the findings exposed that no single factor was responsible for the majority of covariance in the predictor (independent) and criterion (dependent) variables (Podsakoff et al., 2012). Thus, it was concluded that common method variance or bias was not an important concern and was not likely to inflate associations among the variables measured in the current study (Conway and Lance, 2010).

## Analysis and Findings

For assessing and reporting PLS-SEM path model results, the current study used a two-step process as suggested by Anderson and Gerbing (1984), because it is comprised of (a) an assessment of measurement model, also known as an outer model, and (b) an assessment of structural model, also called as an inner model (Hair et al., 2017).

### Assessment of Significance of the Measurement (Outer) Model

In the measurement, the validity and reliability of the constructs were analyzed. For the reliability of each item, the factor loadings were gathered, and for construct reliability, the composite reliability (CR) values were met. Both reliability components exceeded the minimum criteria and show an adequate level of reliability (Hair et al., 2017). To determine the convergent validity, this author used the average variance extracted (AVE) of each of the latent constructs as recommended by Fornell and Larcker (1981). The AVE values of all latent constructs of the current study exceeded the minimum criteria of 0.50 thus, the current study exhibited the adequate convergent validity of the latent constructs (Chin, 1998). Table 1 presented the loadings, composite reliability, and average variance extracted values of each construct.

**TABLE 1:** Loadings, composite reliability, and average variance extracted.

Latent constructs and indicators	Loadings	(AVE)	(CR)
Strategic orientation		0.571	0.889
SO1	0.761		
SO2	0.784		
SO3	0.786		
SO4	0.786		
SO5	0.725		
SO6	0.689		
Organizational culture		0.591	0.961
OC1	0.781		
OC10	0.765		
OC11	0.794		
OC12	0.806		
OC13	0.791		
OC14	0.773		
OC15	0.791		
OC16	0.677		
OC18	0.646		
OC2	0.813		
OC3	0.806		
OC4	0.764		
OC5	0.798		
OC6	0.775		
OC7	0.769		
OC8	0.761		
OC9	0.744		
Internal Market orientation		0.651	0.881
IMO1	0.715		
IMO2	0.839		
IMO3	0.866		
IMO4	0.799		
Organizational commitment		0.631	0.922
OCT1	0.667		
OCT2	0.742		
OCT3	0.841		
OCT4	0.861		
OCT5	0.855		
OCT6	0.786		
OCT7	0.790		
Organizational performance		0.509	0.930
OP1	0.551		
OP10	0.762		
OP11	0.756		
OP12	0.774		
OP13	0.784		
OP2	0.609		
OP3	0.521		
OP4	0.672		
OP5	0.696		
OP6	0.764		
OP7	0.757		
OP8	0.791		
OP9	0.762		

Henseler et al. (2015) proposed the heterotrait-monotrait ratio of correlations (HTMT) as the modern approach (new criterion) to ascertain the discriminant validity. According to Henseler et al. (2016), “the HTMT is an estimate for the factor correlation, thus to clearly discriminate between two factors, the HTMT should be significantly smaller than one” (p. 11). The current study used the HTMT method to ascertain the discriminant validity issues. According to the Henseler et al. (2015), the HTMT estimates the factor correlation, and in order to distinguish between two factors (constructs), the HTMT value should be lower than 1, which indicates that the correlation between the two factors (constructs) is diverse from one another, hence it should differ. Moreover, if the HTMT value is greater than the threshold, this indicates a lack of discriminant validity. Table 2 presents the HTMT values for the discriminant validity of the current study constructs. From the table, it was found that all the HTMT values were lower than the threshold value of 1 and the threshold value of 0.85 (Kline, 2011; Henseler et al., 2015). Hence, it represents the overall acceptable constructs discriminant validity.

**TABLE 2:** HTMT correlation matrix for discriminant validity.

	IMO	OC	OCT	OP	SO
**Int mkt orientation**					
Org. culture	0.543				
Org. commitment	0.668	0.820			
Org. performance	0.566	0.600	0.642		
Str. orientation	0.802	0.664	0.832	0.614	–

### Assessment of Significance of the Structural (Inner) Model

The current study used the standard bootstrapping procedure with 5,000 bootstrap samples and 260 cases to estimate the significance of the path coefficients, according to Hair et al. (2017). Table 3 provides the estimates of the structural model direct relationships and the mediated relationships.

**TABLE 3:** Hypotheses testing.

Hypothesis	Relationship	Standard beta	Standard error	*t*-value	*p*-value	Decision	LL	UL
H1	SO → OP	0.129	0.078	1.656	0.049	**Yes**	0.023	0.269
H2	OCU → OP	0.263	0.092	2.870	0.002	**Yes**	0.084	0.392
H3	IMO → OP	0.000	0.048	0.003	0.499	No	0.000	0.000
H4	SO → OCO	0.397	0.054	7.411	0.000	**Yes**	0.312	0.482
H5	OCU → OCO	0.493	0.047	10.456	0.000	**Yes**	0.417	0.571
H6	IMO → OCO	0.070	0.048	1.453	0.073	**Yes**	0.007	0.163
H7	OCO → OP	0.200	0.104	1.919	0.028	**Yes**	0.040	0.388
H8	SO → OCO → OP	0.079	0.039	2.02	0.044	**Yes**	0.005	0.161
H9	OCU → OCO → OP	0.098	0.048	2.029	0.043	**Yes**	0.008	0.202
H10	IMO → OCO → OP	0.014	0.014	1.025	0.306	No	–0.005	0.047

We first tested the direct relationship of strategic orientation, organizational culture, and internal marketing orientation on organizational performance. The results indicate that SO (β = 0.129, *p* < 0.01) and organizational culture (β = 0.263, *p* < 0.01) were positively related to performance while internal marketing orientation (β = 0.000, *p* > 0.05) was not significant. Thus, H1 and H2 were supported while H3 was not supported. Next, we tested the effect of strategic orientation, organizational culture, and internal marketing orientation on organizational commitment and then the effect of organizational commitment on performance. Strategic orientation (β = 0.397, *p* < 0.01), organizational culture (β = 0.493, *p* < 0.01) and internal marketing orientation (β = 0.070, *p* < 0.1) were all positively related to organizational commitment, while organizational commitment was also positively related to performance (β = 0.200, *p* < 0.05), which supported H4, H5, H6, and H7. The *R*^2^ for organizational commitment was 0.459 and for performance was 0.709. The modeled variables explained 45.9% of the variance in organizational commitment and 70.9% of the variance in performance.

The mediation effect was tested following the guidelines of Preacher and Hayes (2004) and used the bootstrapping indirect effect method. Mediation is confirmed if 0 does not straddle the upper and lower limit of the bias-corrected bootstrapped confidence intervals. As shown in Table 3, the mediation of SO→OCO→Performance (β = 0.079, *p* < 0.01) and OC→OCO→Performance (β = 0.098, *p* < 0.01) were significant while IMO→OCO→Performance (β = 0.014, *p* > 0.05) was not significant. Thus, H8 and H9 were supported while H10 was not supported.

## Contributions and Implications

### Discussion

The first research objective of this study was “to explore the impact of strategic orientation, organizational culture and internal marketing orientation on organizational performance in the six large banks of Pakistan.” Corresponding to the first research objective, three hypotheses (H1, H2, H3) were formulated whereby strategic orientation and organizational performance (H1) concluded with a highly significant relationship. In parallel, hypothesis H2 also explained the significant direct relationship between organizational culture and organizational performance. In contrast, H3 pertaining to Internal marketing orientation and organizational performance failed to report any statistical support.

An important explanation for the strategic orientation and organizational performance relationship could be traced from past studies which confirmed the significant positive relationship in strategic orientation and organizational performance studies (Escribá-Esteve et al., 2008; Pleshko and Nickerson, 2008; Weinzimmer et al., 2012, 2013; Storey and Hughes, 2013; Chahal et al., 2016; Weinzimmer and Robin, 2016). For instance, the study of Chahal et al. (2016) investigated the impact of strategic orientation on the business performance of small and medium enterprises (SME’s) in India. Furthermore, the results of hypothesis H2 agreed with the results of past studies that investigated the organizational culture and organizational performance relationship in different work settings and found a significant positive organizational culture and organizational performance relationship (Aktaş et al., 2011; Storey and Hughes, 2013; Acar and Acar, 2014; Pinho et al., 2014; Graca and Arnaldo, 2016). For instance, Graca and Arnaldo (2016) examined the culture-performance relationship in the biggest dairy company in the Iberian Peninsula.

Additionally, the current study’s result regarding the organizational internal marketing orientation and performance relationship was not consistent with past studies investigating the internal marketing-performance relationship or internal market orientation-performance relationship in a different work setting. The past studies found significant and positive results in the internal marketing orientation-performance relationship (Theodoridis and Panigyrakis, 2011; Zaman et al., 2012; Martin and To, 2013; Yu et al., 2016). For instance, Yu et al. (2016) study examined the internal marketing orientation and organizational performance relationship as a management tool to smooth the employer–employee relationship. The current study’s findings revealed that the banking sector’s internal marketing practices did not directly enhance the bank’s performance. One possible reason is that the Pakistani banks focus on profit maximization, and they have lesser concern for employees because their IM practices do not directly enhance their profitability. The other strategic measures of the banks enhance their profitability. This finding would encourage future researchers to investigate this issue further. In summary, this study explained that proper adoption of strategic orientation, organizational culture, and internal marketing orientation would lead toward enhanced organizational performance. It was supported that these strategic attributes worked as a predictor of organizational performance except internal marketing orientation.

**FIGURE 1:**
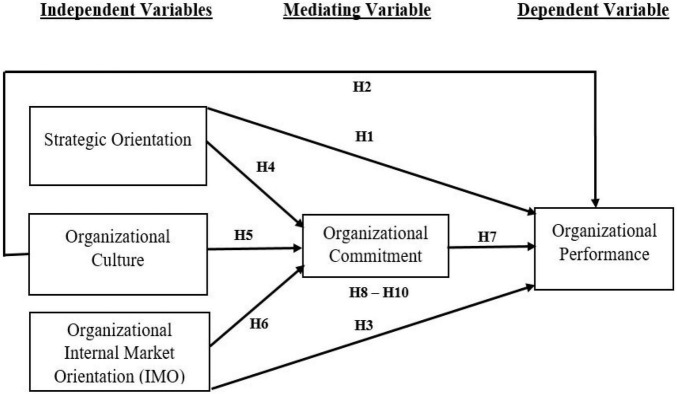
Research model.

The second research objective of the study was “to investigate the impact of strategic orientation, organizational culture and internal market orientation on organizational commitment in the six large banks of Pakistan.” Overall, the second research objective was linked by three hypotheses (H4, H5, H6). Hypothesis H4 concerned the significant direct relationship between strategic orientation and organizational commitment, while hypothesis H5 focused on the significant positive relationship between organizational culture and organizational commitment (Figure 1). The remaining hypothesis, H6 concerned the significant positive relationship between internal market orientation and organizational commitment. For hypothesis H4, the present study found statistical support for a significant positive relationship established between strategic orientation and organizational commitment. The hypothesis H4 results seem to be consistent with other research which found a similarly significant and positive relationship between strategic orientation and organizational commitment (Bhatnagar, 2007; Lee et al., 2010; Rod and Ashill, 2010; Kidombo et al., 2012; Ifie, 2014; Choi and Yoon, 2015).

Another important finding was a significant and positive relationship between organizational culture and commitment, supporting hypothesis H5. The results of hypothesis H5 were in line with those of previous studies that examined the organizational culture and organizational commitment relationship (Pathardikar and Sahu, 2011; Ghorbanhosseini, 2013; Shurbagi and Zahari, 2014; Neelam et al., 2015; Phetkaew, 2015; Majid et al., 2016; Yildirim et al., 2016). This explained that strategic orientation and organizational culture are significant predictors of organizational commitment. Hypothesis H6 concerned the positive effect of organization‘s internal market orientation on organizational commitment, and the finding showed a significant positive relationship between internal marketing orientation and organizational commitment. This finding shows that internal marketing orientation is related to the organizational commitment in the six large banks of Pakistan. The studies revealed that high organizational commitment resulted in decreased employee turnover, absenteeism, ineffectiveness, critical financial losses, and vice versa (Lopes and Kachalia, 2016).

The third research objective of the study was “to determine the relationship between organizational commitment and organizational performance in the six large banks of Pakistan.” Consistent with the third research objective, hypothesis H7 was analyzed and indicated a positive relationship between organizational commitment and organizational performance in the banking sector. This result may be explained by the fact that organizational commitment is a good predictor of organizational performance. This positive finding of the third research objective seems to be consistent with other researchers who found the positive relationship between organizational commitment and organizational performance (Ali et al., 2010; Choi and Yoon, 2015; Omira, 2015; Zincirkiran et al., 2015). For instance, the study of Zincirkiran et al. (2015) examined the effect of organizational commitment on organizational performance in the Turkish health sector.

The fourth research question of the study gave rise to the possible mediating effect of organizational commitment. The equivalent fourth research objective was “to examine the mediating effect of organizational commitment on the relationship between strategic orientation, organizational culture, internal marketing orientation and organizational performance in the six large banks of Pakistan.” Related to this research objective, three hypotheses were analyzed in the current study (H8, H9, H10). The PLS-SEM mediation results provided statistical support for two of the study’s hypothesized relationships (H8 and H9). As hypothesized in the current study, the PLS-SEM results showed that organizational commitment positively mediated the relationship between strategic orientation and organizational performance and between organizational culture and organizational performance.

The positive mediation results of the current study are also in agreement with prior empirical studies, which confirmed the positive relationship between strategic orientation and organizational commitment (Ifie, 2014; Choi and Yoon, 2015), organizational culture, and organizational commitment (Neelam et al., 2015; Yildirim et al., 2016), and organizational commitment and organizational performance (Cura, 2014; Chai et al., 2016) in separate studies. In addition, one unanticipated finding was that hypothesis H10 was not supported due to the insignificant mediating effect of organizational commitment between organization’s internal marketing orientation and organizational performance. It is somewhat surprising and thus requires further empirical attention for confirmation. Though the present study found results contradicting our assumptions, yet still they were parallel to the findings of Awwad and Agti (2011) and Zaman et al. (2012), who also reported no mediation of organizational commitment between internal marketing (predictor) and market orientation and organizational citizenship behavior (criterion) studied in the banking sector (Table 4).

**TABLE 4:** Profile of respondents.

Characteristics	Frequency	Percentage
**Gender**		
Male	197	75.8
Female	63	24.2
**Age**		
21–30 Years	77	29.6
31–40 Years	81	31.2
41–50 Years	78	30.0
51–60 Years	24	9.2
**Qualification**		
Bachelor	60	23.1
Masters	26	10.0
Banking diploma	169	65.0
Non-banking qualification	5	1.9
**Job position**		
Branch Manager	260	100

### Theoretical Implications

The current study had two underlying theoretical perspectives: RBV theory of the firm and SET. The emphasis of SET is on the exchange relationship between employer and employee which starts with one person giving something to another, and then another will also reciprocate the exchange, which highlights the sense of belonging and gratitude between employer and employee. RBV theory explains that organizations should adopt unique internal resources in order to gain a competitive advantage and organizations should also focus on their internal resources, e.g., human resources, internal culture, or procedure (Barney, 1991; Bakar and Ahmad, 2010; Uzkurt et al., 2013; Bertram, 2016).

Previous studies were mixed and contradictory, which the present study has attempted to address through adding novel empirical insights toward the body of knowledge on the tested relationships. The current study found that all strategic attributes act as positive actions from the employer, and employees reciprocate in the form of high commitment and organizational performance. Moreover, the current research explained the relationship between strategic attributes and organizational performance and commitment.

### Practical Implications

This research significantly contributed to management actions that can be practically implemented, especially for Pakistani banking organizations, financial regulatory authorities, and bank managers. Most importantly, it highlighted a need for banking organizations to pay more attention to the employee value proposition and internal marketing practices in Pakistan’s banking sector. Besides, banking is no longer the preferred choice among business graduates as they are moving toward the fast-moving consumer goods FMCG sector and IT services sector, which is not a positive sign for banking organizations, and could affect future organizational performance. Therefore, this study highlighted the importance of organizational behavior regarding internal market orientation and its relationship with organizational performance. This study has found support for the mediating role of organizational commitment in the relationship between strategic attributes and organizational performance. These findings have offered valuable insights, particularly for Pakistani banking organizations and bank managers, and emphasized the requirement of organizational commitment for organizations to be strategically positioned. The findings of the study have educated that businesses in the financial sector such as banks have to focus on employees‘ giving shared vision, clear direction and effective plans to boost their commitment and thereby improving organizational performance. Training interventions can be helpful in regard (Mihardjo et al., 2020). Accordingly, the findings also imply that management practitioners in banks develop an environment whereby employees experience a sense of genuinity, welfare, organizational concern for their needs, importance for their work, and individual values.

The findings of the current study supported the need for bank management and bank managers to understand the importance of a strong bank culture. It supported managers in changing their bank management lifestyles and for organizations to create job satisfaction among employees, which will help the employees remain with their bank and not job search. It encouraged the view that employers and employees both can easily maintain a work-life balance and be committed and productive to the organization. It is s also essential to consider the vital role of a strong culture in banks that can be nurtured through employee development activities. Latest banking technology and employer branding activities should also be undertaken as this study supports that these will increase the commitment level and organizational performance (Badar, 2011).

For practitioners, the implications of the findings should be clear. An organization that wishes to enhance the organizational performance should pay attention to its strategic attributes, including strategic orientation, organizational culture, and organizational internal market orientation, as it can be a crucial enabler for both the organizations and authorities for removing the barriers of achieving high performances. In particular, the results of this research showed that adoption of strategic attributes fostered performance and innovation, which implied that organizations must make efforts to highlight the importance of strategic attributes.

## Conclusion

Conclusively, the current study attempted to respond to several gaps in the literature pertaining to strategic attributes, organizational commitment and organizational performance. The study empirically investigated the effects of strategic attributes (strategic orientation, organizational culture, internal marketing orientation, and organizational commitment) on organizational performance, followed by the mediating role of organizational commitment across the six large banks of Pakistan. The study has contributed theoretically to the pertinent literature whilst underlining important implications for managerial practice on how financial institutions such as banks can capitalize upon their strategic attributes to harness organizational commitment, which further boosts organizational performance.

## Limitations and Suggestions for Future Study

The current study’s objectives have been achieved to a greater extent. The findings provided theoretical, practical, and social contributions. Nevertheless, it needs to be mentioned that there were some limitations of the current study which further highlighted the scope for future studies. The current study was only focused on the six large banks in the Pakistani banking sector, which may require further study to be able to make generalizations both inside and outside of Pakistan, such as to other services organizations and smaller banks inside Pakistan, and to banks and other service organizations outside Pakistan. These may provide areas for fruitful future research to be conducted. Other services organizations would include insurance companies, hotels, and education. Accordingly, the study also recommends that future scholars consider testing the same framework across different geographical settings to generalize the results.

Also, the current study was a cross-sectional study, and data were collected and analyzed at one point in time. Thus, only a relatively small population was studied. Therefore, future researchers may want to conduct a comparable but longitudinal study to analyze the constructs over a prolonged time period for receptive confirmation of the hypothesized relationships of the present study. It is relatively complex to generalize the findings of the current study because the data were collected from only the six large banks of Pakistan located in the five main cities of Pakistan. Accordingly, it could be suitable to study the other banks functioning in Pakistan and in other cities to generalize the results to the entire Pakistani banking sector.

Finally, it might be beneficial to study the banks and compare the findings with the other financial institutions operating in Pakistan for a comprehensive understanding of the entire financial services sector and organizational performance prospects.

## Data Availability Statement

The raw data supporting the conclusions of this article will be made available by the authors, without undue reservation.

## Ethics Statement

The studies involving human participants were reviewed and approved by Pakistan Ethical Board. The patients/participants provided their written informed consent to participate in this study.

## Author Contributions

All authors listed have made a substantial, direct, and intellectual contribution to the work, and approved it for publication.

## Conflict of Interest

The authors declare that the research was conducted in the absence of any commercial or financial relationships that could be construed as a potential conflict of interest.

## Publisher’s Note

All claims expressed in this article are solely those of the authors and do not necessarily represent those of their affiliated organizations, or those of the publisher, the editors and the reviewers. Any product that may be evaluated in this article, or claim that may be made by its manufacturer, is not guaranteed or endorsed by the publisher.
