# Supplementary material for: Strategic Attributes and Organizational Performance: Toward an Understanding of the Mechanism Applied to the Banking Sector
Source: Front Psychol. 2022 May 12;13:855910. doi: 10.3389/fpsyg.2022.855910 (PMC9133961; doi:10.3389/fpsyg.2022.855910)
Supplement: Supplementary file 1 [file Data_Sheet_1.docx]

**Appendix A: Adapted Questionnaire Items**

*Organizational Performance*

| The level of our customer satisfaction with our bank services. |
| --- |
| The reactivation of inactive (dormant) bank accounts. |
| The customer service delivery in our bank (branch). |
| The customer relationship management in our bank (branch). |
| The branch reputation of our bank in the business environment. |
| The rate of speed of services in our bank (branch). |
| The operating cost of providing services to customers in our bank (branch). |
| The error of operational processes in our bank (branch). |
| The new services / products introduced in our bank (branch). |
| The market share in public and private banking sector business. |
| The number of our bank (branch) performing loans. |
| The yearly profit performance of our bank (branch). |
| The number of non-performing loans of our bank (branch). |
| The bank deposit liability growth. |
| The number of recovered bad loans of our bank (branch). |
| The fee-based income on transaction services of our bank (branch). |
| The volume of current and saving accounts of our bank (branch). |
| The volume of a fixed deposits of our bank (branch). |
| The financial performance targets achievement by our bank (branch). |
| The operating expenses of our bank (branch). |

*Strategic Orientation*

| There is a shared vision of what the bank will be in the future. |
| --- |
| Our strategic direction is clear. |
| The bank goals and objectives are linked to our mission, vision, and strategy. |
| Short-term thinking does not compromise our long-range vision. |
| We have effective strategic plans. |
| The bank has regular and effective planning processes at all levels. |

*Organizational Culture*

| In our bank (branch) most employees remain highly engaged in their work. |
| --- |
| Information in our bank (branch) is widely shared so that everyone can get the information he or she needs. |
| Teams are the primary building blocks in our bank (branch). |
| Work is organized so that every employee can see the relationship between his/her job and the overall bank goals. |
| Our bank (branch) continuously invests in the skills of employees. |
| Our bank (branch) views the capabilities of people as an important source of competitive advantage. |
| Our bank (branch) has a clear and consistent set of values that govern the way we do business. |
| Our bank (branch) sets a clear agreement about the right way and the wrong way to do things. |
| In our bank (branch), there is a good alignment of goals across levels. |
| In our bank (branch), we respond well to competitors and the day to day changes in the business environment. |
| Different departments of our bank (branch) often cooperate to create change. |
| In our bank (branch), customers’ input directly influences our decisions. |
| In our bank (branch), we encourage direct contact with customers by our employees. |
| In our bank (branch), we view complaints as an opportunity for learning and improvement. |
| In our bank (branch), innovation is encouraged and rewarded. |
| In our bank (branch), there is a clear mission that gives meaning and direction to our work. |
| In our bank (branch), employees understand what needs to be done for us to succeed in the long run. |
| Bank vision creates excitement and motivation for our employees. |

*Organizational Internal Market Orientation*

| The bank for which I work is genuinely concerned with the welfare of all its employees. |
| --- |
| The bank for which I work tries to accommodate different personal needs of all its employees. |
| The bank for which I work does not recognize the importance of its employees. |
| My bank values its employees. |

*Organizational Commitment*

| Employees feel as though their future is intimately linked to that of this bank. |
| --- |
| Employees are happy to make personal sacrifices if it is important for the well-being of the bank (branch). |
| The bonds between this bank (branch) and its employees are weak. |
| In general, employees are proud to work for this bank (branch). |
| Employees often go above and beyond the call of duty to ensure the well-being of the bank (branch). |
| Our employees have little or no commitment to this bank (branch). |
| It is clear that our employees are fond of this bank (branch). |
